# Increased risk of a suicide event in patients with primary fibromyalgia and in fibromyalgia patients with concomitant comorbidities: A nationwide population-based cohort study

**DOI:** 10.1097/MD.0000000000005187

**Published:** 2016-11-04

**Authors:** Chen-Chia Lan, Chun-Hung Tseng, Jiunn-Horng Chen, Joung-Liang Lan, Yu-Chiao Wang, Gregory J. Tsay, Chung-Yi Hsu

**Affiliations:** a Department of Psychiatry, Taichung Veterans General Hospital; b School of Medicine, China Medical University; c Department of Neurology; d Division of Immunology and Rheumatology, Department of Internal Medicine, China Medical University Hospital, Taichung, Taiwan; e Rheumatology Research Laboratory, China Medical University; f Management Office for Health Data, China Medical University Hospital; g Graduate Institute of Clinical Medical Science, China Medical University, Taichung, Taiwan.

**Keywords:** anxiety, depression, Fibromyalgia, headache, sleep disorder, suicide event

## Abstract

An increased risk of suicide ideation and death has been reported in patients with fibromyalgia. This study aimed to evaluate the risk of a suicide event in patients with primary fibromyalgia and in fibromyalgia patients with comorbidities. We used the Longitudinal Health Insurance Database, a subset of the national insurance claim dataset, which enrolled 1 million Taiwanese people from 2000 to 2005, to identify 95,150 patients with incident fibromyalgia (ICD-9-CM 729.0–729.1) and 190,299 reference subjects matched by sex, age, and index date of diagnosis, with a mean of 8.46 ± 2.37 years of follow-up until 2011. The risk of a suicide event (ICD-9-CM, External-Cause Codes 950–959) was analyzed with a Cox proportional hazards model. Stratification analysis was performed by separating fibromyalgia patients and reference subjects with respect to each comorbidity to determine the risk of suicide in fibromyalgia patients with or without comorbidity relative to subjects who had neither fibromyalgia nor comorbidity. In this Taiwanese dataset, there were 347 suicide events in patients with fibromyalgia (4.16 per 10^4^ person-years) and 424 in matched reference subjects (2.63 per 10^4^ person-years) with a significant crude hazard ratio (HR) of 1.58 (95% confidence interval [CI] 1.38–1.83) and an adjusted HR of 1.38 (95% CI 1.17–1.71) for fibromyalgia patients relative to the matched reference subjects. According to the 2 × 2 stratification analysis, we found that fibromyalgia patients without comorbidity had an independent but mild risk of a suicide event with adjusted HRs ranging from 1.33 to 1.69 relative to subjects with neither fibromyalgia nor comorbidity. Meanwhile, fibromyalgia patients with comorbidity led to a markedly enhanced risk of a suicide event relative to the matched reference subjects, with adjusted HRs ranging from 1.51 to 8.23. Our analysis confirmed a mild-to-moderate risk of a suicide event in patients with primary fibromyalgia. Attention should be paid to the prevention of suicide in fibromyalgia patients with concomitant comorbidities.

## Abstract 2

Supplemental Digital Content is available in the text

## 1 Introduction

Fibromyalgia is characterized by chronic widespread pain, unrestorative sleep, overwhelming fatigue, emotional alteration, and cognitive dysfunction with impaired daily social functioning and a reduced quality of life.^[1]^ Although the etiology of fibromyalgia is unknown, the pain pattern is described as hyperalgesia and allodynia.^[2]^ There is a higher prevalence of fibromyalgia in women than in men.^[1,3]^ Based on the 1990 classification criteria of the American College of Rheumatology for fibromyalgia, the worldwide prevalence is ∼2% to 4%,^[1–3]^ whereas the prevalence is lower in the Asian population.^[4,5]^ In contrast, recent population-based studies using the modified 2010 classification criteria of the American College of Rheumatology^[6]^ reported a higher prevalence (∼5–7%) of fibromyalgia.^[7,8]^

The most common risk factors for suicidality across various countries include being female, young, or unmarried; having a lower level of education; and having a mental disorder.^[9]^ Predictors of suicidal ideation and suicide attempts include comorbid depression, sleep alteration, and insomnia.^[10]^ In a Canadian study, an increased frequency of suicidal ideation and attempts were correlated with chronic, widespread noncancer-related pain.^[11]^ A positive association between chronic pain and suicidal behavior and death is suggested.^[12,13]^

Generalized pain, depressive symptoms, and nonrestorative sleep, all of which often occur in fibromyalgia patients,^[6]^ are risk factors for suicidality. Female patients with a confirmed or possible diagnosis of fibromyalgia have a 10-fold increase in suicide frequency based on a Danish observational study in the referral hospital.^[14]^ Comorbidities are commonly associated with an increased risk for suicide in fibromyalgia patients, which can be explained by concomitant psychiatric disorders including depression.^[14–16]^ Evidence indicating whether there is an independently causal relationship between fibromyalgia and suicide is still limited.

The present study was designed to study whether patients with primary fibromyalgia have a higher risk of a suicide event by using the Taiwanese National Health Insurance Research Database (NHIRD). The risk of a suicide event associated with concomitant comorbidities of medical and mental disorders in fibromyalgia patients was also investigated.

## 2 Methods

### 2.1 Data source

The National Health Insurance (NHI) began in 1995 and covers >99% of Taiwan's population. The NHIRD which belongs to the NHI was our main data source for this population-based cohort study. This dataset contains information from registration and reimbursement claims. Previous report has confirmed the accuracy and validity of diagnoses in the NHIRD.^[17]^ Our database containing medical reimbursement claims from 1996 to 2011 for 1 million people randomly selected from the NHIRD.^[18]^ This database which can represent the whole population was released by the NHI as the Longitudinal Health Insurance Database (LHID). All data were de-identified and subsequently analyzed anonymously for personal data protection when the data were released for research. The age and sex of patients in the LHID and NHIRD did not differ significantly.^[17,18]^ The ethical review board of China Medical University in Taiwan approved his study (CMU-REC-101-012).

### 2.2 Criteria for selecting subjects

**Figure 1:**
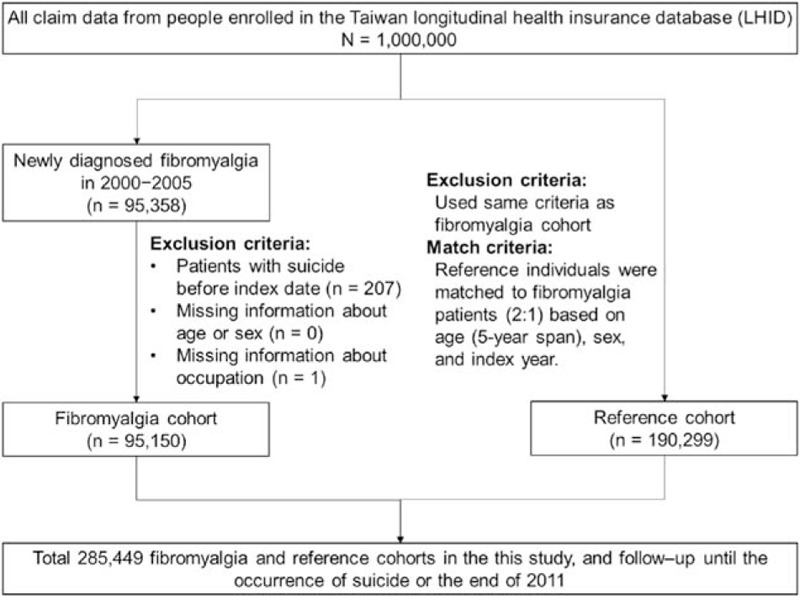
Flowchart of the study design.

Diseases of interest in the NHIRD are coded using the International Classification of Diseases, 9th Revision, Clinical Modification (ICD-9-CM). To prevent medical fraud by overbilling for health care or inappropriate charges based on unconfirmed diagnoses, the disease coding is strictly regulated by the NHI. The coding errors, misdiagnosis, and inappropriate treatment were carefully avoided to support the reliability of LHID data for exploring the risk of suicidal behavior in fibromyalgia patients. The study design is shown in Fig. 1. Among the patients in the LHID, 95,358 cases of incident fibromyalgia were identified by using cases in which 3 sequential clinical diagnoses of ICD-9-CM 729.0–729.1 at out-patient clinics were made during the period from 2000 to 2005. The index date for that individual in the LHID was defined when each patient's first fibromyalgia diagnosis was made.^[19]^ All subjects were followed from the index date until they were censored, withdrew from the database, or until the end of the observation period on 31 December 2011. Patients were excluded from the study (n = 208) if their records had missing data on age or sex or were diagnosed with a suicide event before the diagnosis of fibromyalgia. Excluding these 208 patients, the 95,150 remaining patients with fibromyalgia were designated as the case cohort. The initial pool for the reference cohort (n = 761,001) consisted of the rest of the study population after excluding the identified fibromyalgia patients, those who had a diagnosis of fibromyalgia but did not satisfy the inclusion criteria due to <3 visits, those who were diagnosed fibromyalgia before or after this period of time, those who had a suicide event before the index date, and those cases with missing data. Patients with fibromyalgia were matched at baseline by age, sex, and index date at a 1:2 ratio with a reference cohort of nonfibromyalgia subjects in the LHID (n = 190,299; only 1 nonfibromyalgia reference subject did not qualify for matching criteria).

### 2.3 Primary outcome and relevant variables

The primary outcome was the occurrence of a suicide event or self-inflicted injury with clinical diagnosis (ICD-9-CM, E-Codes: 950–959) during the study period (2000–2011). Therefore, the primary outcome of a suicide event included completed suicide, a suicide attempt, and nonsuicidal self-injury.

Relevant variables included age, sex, and comorbidities, including diabetes mellitus (ICD-9-CM 250), disorder of lipoid metabolism (ICD-9-CM 272), hypertensive disease (ICD-9-CM 401–405, except congestive heart failure), congestive heart failure with or without renal disease (ICD-9-CM 402.01, 402.11, 402.91, 404.01, 404.03, 404.11, 404.13, 404.91, 404.93, 428.0), cerebrovascular disease (ICD-9-CM 430–438), irritable bowel syndrome (ICD-9-CM 564.1), chronic liver disease and cirrhosis (ICD-9-CM 571), headache including tension type and migraine headache (ICD-9-CM 307.8, 339.1, 346), depressive disorders or reaction (ICD-9-CM 296.2–296.3, 300.4, 311), anxiety states or phobic, obsessive-compulsive, somatoform disorders (ICD-9-CM 300.0, 300.2, 300.3, 308.3, 308.91), and functional or organic sleep disorder (ICD-9-CM 307, 327, 780.5). All comorbidities were defined before the index date of fibromyalgia diagnosis.^[19]^

### 2.4 Statistical analysis

The differences between the case cohort of fibromyalgia patients and the reference cohort in demographic characteristics and comorbidities were tested with a chi-square test for categorical variables or Student's *t*-test for continuous variables. The sex-, age-, and comorbidity-specific incidence rates (per 10^4^ person-years) of a suicide event between the case and reference cohorts were compared. Person-years were calculated from the index date to the censored date of a suicide event, the date of withdrawal from follow-up, or the end of 2011. The standardized prevalence rate of fibromyalgia was calculated as the prevalence of fibromyalgia cases in 2003 divided by the expected number of fibromyalgia cases. Meanwhile, the standardized incidence rate of fibromyalgia was calculated as the observed newly diagnosis of fibromyalgia cases in 2003 divided by the expected number of fibromyalgia cases. The expected number of fibromyalgia cases was obtained from the product of national gender-specific, age-specific incidence rates from the Taiwanese population^[18,20]^ and the World Health Organization ^[21,22]^ in 2000, respectively.

Differences in the cumulative incidence of suicide events between cohorts, plotted as Kaplan–Meier survival curves, were tested by a log-rank test. The Cox proportional hazard model was used to estimate the hazard ratios (HRs) and 95% confidence intervals (CIs) for a suicide event in fibromyalgia patients relative to reference subjects. Multivariate adjusted HRs (aHRs) were derived by adjusting for age; sex; occupation status; monthly income level; comorbidities of diabetes, hypertension, hyperlipidemia, congestive heart failure, cerebrovascular disease, irritable bowel syndrome, chronic liver disease, headache, sleep disorder, anxiety, and depression; and the use of nonsteroid anti-inflammatory drugs (NSAIDs). Stratification analysis with respect to (1) sex, (2) age, (3) occupation status, (4) monthly income level, (5) the presence or absence of comorbidity, and (6) NSAID use, respectively, was further used to compare the risk of a suicide event in patients with fibromyalgia. Age subgroups were stratified into <35 years, 35 to 65 years, and >65 years. A 2 × 2 stratification analysis was performed by separating fibromyalgia patients and reference subjects with respect to each comorbidity to determine the risk of suicide in fibromyalgia patients with/without comorbidity relative to subjects who had neither fibromyalgia nor comorbidities. A *P*-value <0.05 was considered significant in 2-tailed tests.^[20]^ All calculations were performed using SAS version 9.1 (SAS Institute Inc., Cary, NC).

## 3 Results

From the LHID, we identified 95,150 fibromyalgia patients as the case cohort and 190,299 matched subjects without fibromyalgia as the reference cohort (1:2 match ratio), with a mean 8.46 ± 2.37 years of follow-up (2,443,463 person-years in total). The age- and gender-standardized prevalence and incidence rates of fibromyalgia are 5.84% and 2.02 per 10^2^ person-years, respectively, for the Taiwanese population ^[18,20]^ and 5.56% and 1.92 per 10^2^ person-years, respectively, for the global population according to the World Health Organization.^[21,22]^

The mean age of the fibromyalgia cohort was 45.8 ± 17.2 years, with a female-to-male ratio of 6:4 (Table 1). With stratification according to age, occupation status, or monthly income, the highest proportion of fibromyalgia patients were in the 35- to 65-year-old subgroup (56.4%), were office workers (50.6%) and had a monthly income of 15,000 to 25,000 Taiwanese dollars (TWD) (56.2%). In keeping with a previous report, fibromyalgia patients had a significantly higher prevalence of comorbidities,^[23]^ including diabetes, hypertension, hyperlipidemia, congestive heart failure, cerebrovascular disease, depression, anxiety, irritable bowel syndrome, headache, sleep disorder, and chronic liver disease relative to nonfibromyalgia subjects.

**Table 1:**
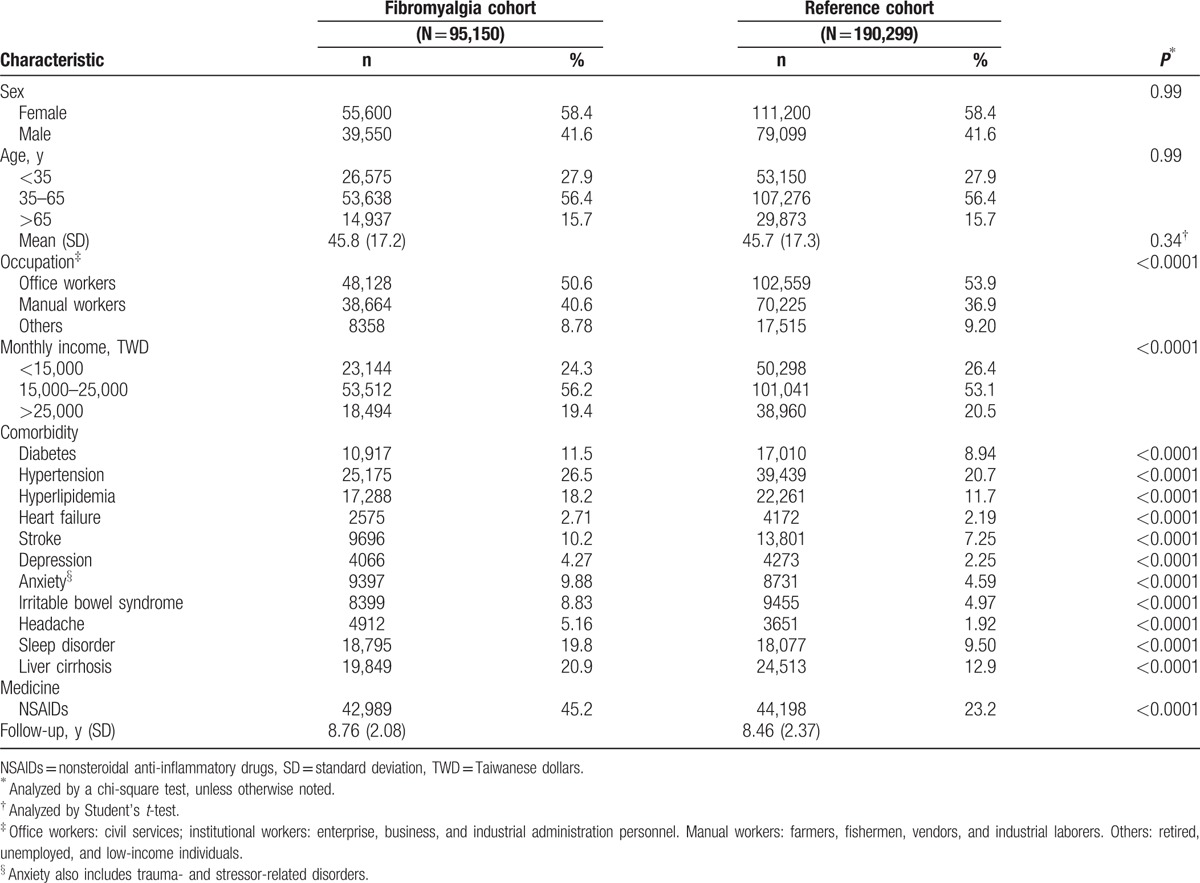
Comparison of the demographics and comorbidities between fibromyalgia patients and the 1-to-2 matched reference cohorts in our study population.

**Figure 2:**
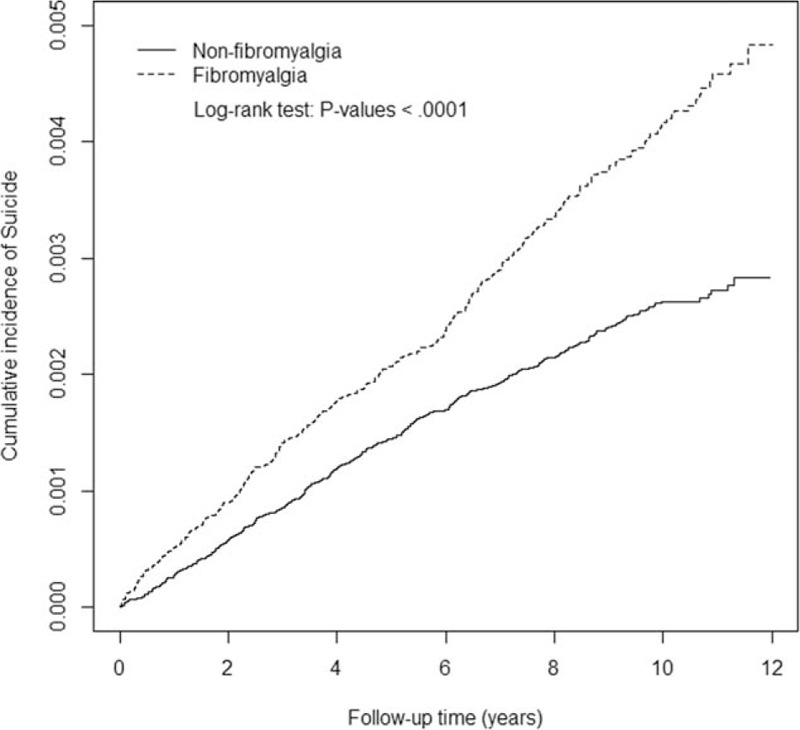
The cumulative incidence of a suicide event in fibromyalgia patients and nonfibromyalgia subjects.

Figure 2 shows the cumulative incidence of suicide events among subjects with and without fibromyalgia during 12 years of follow-up. Among these individuals, 347 fibromyalgia patients (4.16 per 10^4^ person-years) and 424 reference subjects (2.63 per 10^4^ person-years) had suicide events (Table 2). The aHR of a suicide event in fibromyalgia patients relative to the reference subjects was 1.38 (95% CI 1.19–1.60) after adjusting for age, sex, occupation, monthly income, comorbidities, and NSAID use. The risk of suicide was higher in fibromyalgia cohort than nonfibromyalgia cohort regardless of women and men (women: aHR, 1.42, 95% CI, 1.17–1.71; men: aHR, 1.31, 95% CI, 1.03–1.67). The gender difference of risk of suicide in fibromyalgia patients was statistically nonsignificant (aHR of 0.96, 95% CI 0.79–1.17), when we compared the risk of a suicide event in female fibromyalgia patients relative to male patients after adjusting for age, occupation, income, comorbidities, and NSAID use (Supplementary Table 1). However, this risk was significant in the younger subgroup aged ≤65 years (age <35 years: aHR 1.54, 95% CI 1.19–2.00; age 35–65 years: aHR 1.37, 95% CI 1.10–1.72). In addition, there was a noteworthy and significant risk of a suicide event among fibromyalgia patients who were office workers (aHR 1.75, 95% CI 1.39–2.20) and a relatively higher risk in patients with monthly income >TWD 25,000 (aHR 2.17, 95% CI 1.29–3.63) compared with their respective matched counterparts.

**Table 2:**
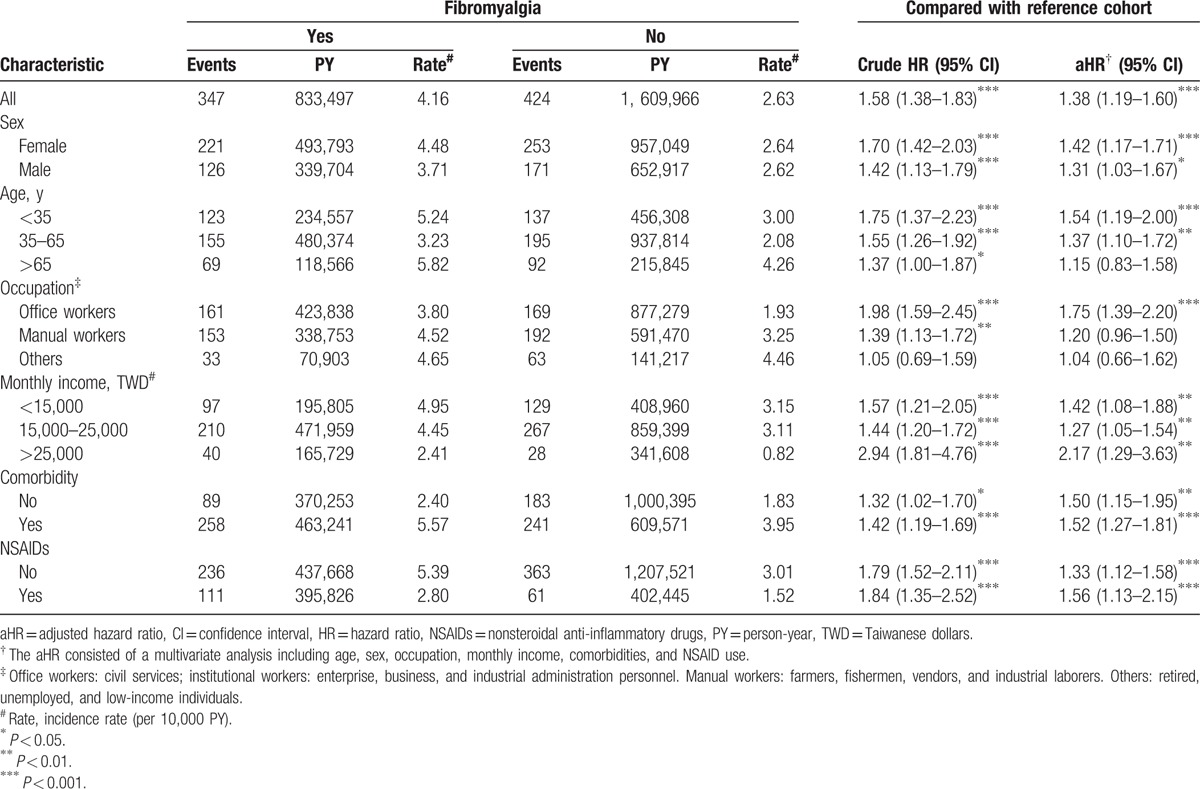
Comparison of the crude and adjusted hazard ratios of a suicide event between fibromyalgia and the 1-to-2 matched reference cohorts with stratification by sex, age, and comorbidity.

**Figure 3:**
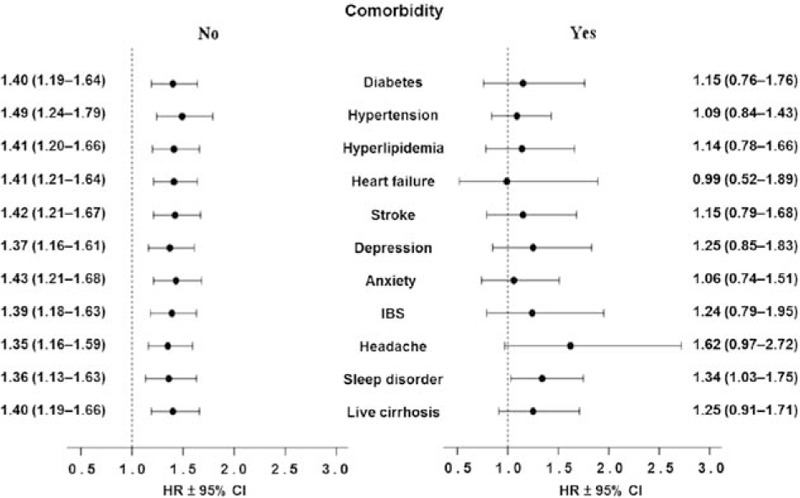
The adjusted hazard ratio (HR) for suicide in fibromyalgia patients compared with the nonfibromyalgia cohort as stratified by comorbidity. Fibromyalgia patients without comorbidity and the reference cohort without comorbidity are compared on the left side, and fibromyalgia patients with a specific comorbidity and the reference cohort with that same comorbidity on the right side. The HR derived by multivariate analysis included age, sex, occupation, monthly income, and NSAID use in addition to the individual comorbidities shown (95% CI = 95% confidence interval, HR = hazard ratio, IBS = irritable bowel syndrome).

Fibromyalgia patients and reference subjects were stratified with respect to each comorbidity. Fibromyalgia patients without comorbidity relative to the matched nonfibromyalgia reference subjects without comorbidity had a significant risk of a suicide event, with aHRs ranging from 1.35 to 1.49 after adjusting for age, sex, occupation, income, comorbidities, and NSAID use (left side of Fig. 3 and Supplementary Table 2). In contrast, the risk of a suicide event in fibromyalgia patients with specific comorbidity was not significant relative to the reference cohort with the same comorbidity, except for sleep disorder after adjusting for age and sex (right side of Fig. 3 and Supplementary Table 2).

**Figure 4:**
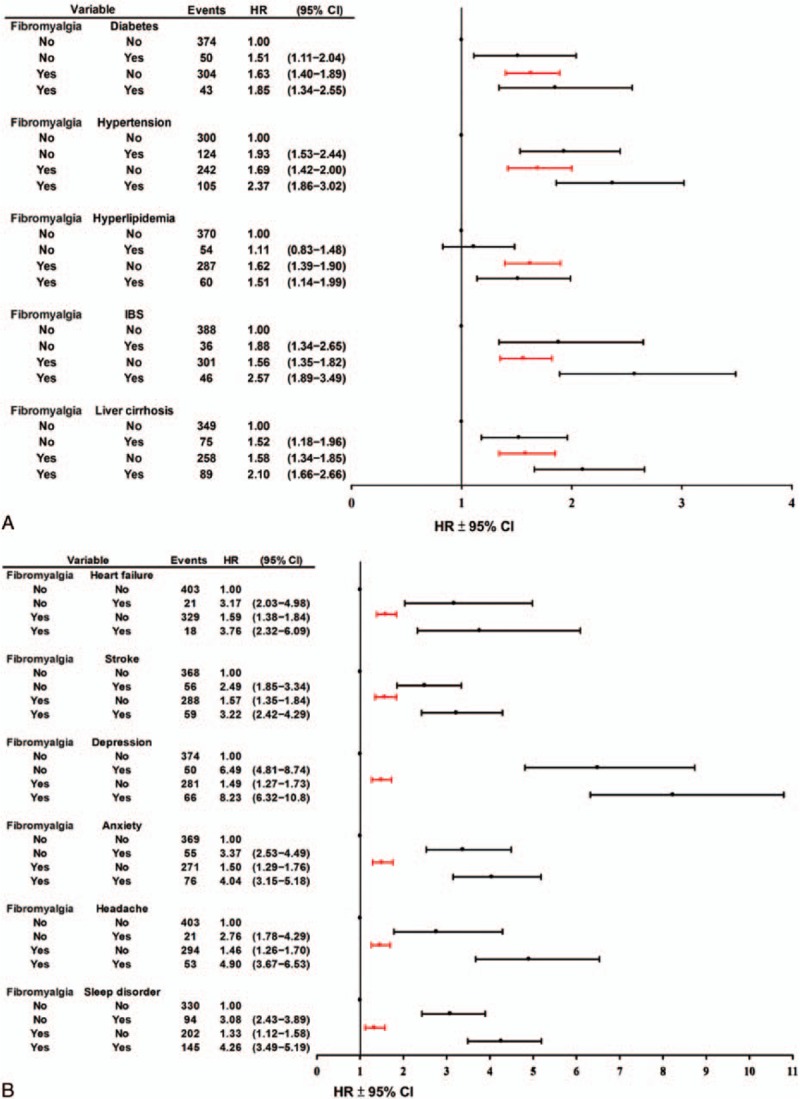
Cox proportional hazard regression analysis for the risk of suicide associated with fibromyalgia with (A) an additional effect of comorbidities including diabetes, hypertension, hyperlipidemia, IBS, and liver cirrhosis and (B) an additional effect of comorbidities including congestive heart failure, cerebrovascular disease (stroke), depression, anxiety, headache, and sleep disorder. In each analysis, subjects with neither fibromyalgia nor respective comorbidity were used as references. The HR derived by the model is adjusted for age and sex. (95% CI = 95% confidence interval, HR = hazard ratio, IBS = irritable bowel syndrome)

After 2 × 2 stratifying the data for fibromyalgia patients and nonfibromyalgia subjects and using subjects with neither fibromyalgia nor comorbidity as the reference, we found the risk of a suicide event in patients with primary fibromyalgia was similar to that of patients with other chronic diseases, such as diabetes, hypertension, irritable bowel syndrome, and liver cirrhosis (Fig. 4A and Supplementary Table 3). In contrast, for comorbidities of congestive heart failure, cerebrovascular disease, depression, anxiety, headache, or sleep disorder, the suicide risk after adjusting for age and sex in fibromyalgia patients without the comorbidity was significantly lower than the respective risk in nonfibromyalgia patients with the comorbidity (Fig. 4B and Supplementary Table 3). As expected, the risk of a suicide event was increased in fibromyalgia patients in combination with an individual comorbidity, except for hyperlipidemia (Fig. 4A, 4B and Supplementary Table 3), which may be ascribed mainly to the addition of biopsychosocial risk from the comorbidity.^[24,25]^

## 4 Discussion

The present study showed an independent but mild to moderate risk of a suicide event in fibromyalgia patients after adjusting for age, sex, occupation status, monthly income, NSAID use, and comorbidities. The level of risk for patients with primary fibromyalgia was similar to that of patients with other chronic diseases,^[24]^ and the risk was lower than that for diseases such as congestive heart failure,^[25]^ cerebrovascular disease,^[26]^ and headache.^[11]^ Furthermore, this risk was much lower than those associated with mental disorders.^[27]^ This finding, obtained using data from the general population of individuals with fibromyalgia, is in contrast to a previous observational study in the referral center that found a 10-fold increased frequency of a suicide event among female fibromyalgia patients.^[14]^ Because fibromyalgia patients frequently have associated comorbidities, the risk of a suicide event in patients with concomitant fibromyalgia is of particular concern and should be considered by health care providers.^[14–16]^

Fibromyalgia patients have a higher prevalence of major depression and panic disorder; further, anxiety significantly predicts physical dysfunction.^[28]^ Anxiety and depression may adversely affect patients’ perception of disease severity and reduce pain tolerance.^[28]^ Furthermore, fibromyalgia patients who seek health care may be more psychiatrically distressed than those who do not.^[28]^ A blunted stress response in fibromyalgia patients with low levels of cortisol in urine after 24 hours and decreased production of cortisol in response to corticotropin-releasing hormone may explain the onset of psychiatric disorders among patients with a debilitating chronic illness.^[29]^ As shown in post-stroke depression, a depressive state that is due to the presence of other diseases may induce suicidal behavior.^[26]^ Fibromyalgia patients with either depression or anxiety had a significant risk of a suicide event after a multivariate analysis, which is compatible with a previous report that examined suicidal behavior among fibromyalgia patients with mood disorders.^[10]^ An mRNA microarray study in individuals with major depressive disorder revealed dysregulation of genes involved in glutamatergic and GABAergic signaling in the cortical and subcortical region,^[30]^ and this alteration in glutamatergic and GABAergic systems may also be found in fibromyalgia patients.^[31]^ Decreased serotonergic neurotransmission in individuals with fibromyalgia ^[32]^ may precipitate a depressive state.^[33]^ These potential mechanisms that increase the risk of suicidal behavior may also explain the increased risk we observed in patients with fibromyalgia.

Stressful circumstances can be perceived as overwhelming triggers for either disease or challenges that can be managed positively without major risk.^[34]^ Environmental stressors, such as inequality of socioeconomic status, may affect both psychiatric disorders and fibromyalgia patients.^[34,35]^ Antonovsky's salutogenic concept of sense of coherence (SOC) was included in the Ottawa Charter of 1986 that not only addressed developing health-care programs but also emphasized promoting health in order to prevent illnesses and negative effects or stress.^[34,36,37]^ Individuals with a strong SOC—including meaningfulness, comprehensibility, and manageability—are more likely to control and cope with problems,^[34]^ and low levels of fatigue, loneliness, and anxiety.^[36]^ On the other hand, patients with lower SOC are more likely to have musculoskeletal symptoms, such as pain; however, low SOC can be improved through coping-skills training and stress management.^[37]^ Fibromyalgia patients reported lower levels of pain and thermal pain sensitivity in the presence of a significant other, compared to when they were alone.^[38]^ Social support that includes supportive, solution-oriented friends, family, and other social networks may help patients cope with chronic stress.

A higher incidence rate of a suicide event in fibromyalgia patients of <35 years of age (5.24 per 10^4^ person-years) than in the reference subjects (3.00 per 10^4^ person-years) was noted. According to most studies, suicide is the second most common cause of death for young people after accidents.^[39]^ SOC was significantly lower in the youngest age group and increased with age.^[35]^ Higher levels of relative deprivation and lower social cohesion may affect mortality trends among young adults.^[40]^ Comorbidities of impulsivity and anxiety that are due to stress or trauma in young people may correlate with an increased frequency of suicidal behavior.^[41]^ Suicide attempts occur more commonly among young women, whereas completed suicide occurs more frequently in middle-aged men.^[42,43]^ Our study confirmed that for individuals aged ≤65 years, an independent risk of suicide in fibromyalgia patients was noted relative to the matched reference cohort.^[44]^ The psychological effects of relative deprivation are not only confined to health care but are also associated with suicide death, acquired immunodeficiency syndrome (AIDS), violence, and cirrhosis of liver.^[40]^ Our finding is relevant to the care of young fibromyalgia patients.

Chronic stress has a strong correlation to socioeconomic differences, due to the increased burden of relative deprivation.^[40]^ An association between wealth and mortality was indicated by a prospective cohort study: a higher risk of mortality was found in the intermediate and lower wealth tertiles, compared to those in the highest wealth tertile.^[45]^ This study further showed a significant risk of a suicide event in fibromyalgia patients who were office workers and a relatively higher risk was noted in those with a monthly income of >TWD 25,000, compared with the respective matched reference cohort. This phenomenon may be ascribed to a higher prevalence of lifetime stress and sleep disorders in individuals with a higher socioeconomic status.^[46]^ Because sedentary occupation leads to metabolic syndrome and diabetes, either of which can also result in physiological pain, depression, and suicide,^[47,48]^ introducing health behaviors to reducing sedentary behavior was suggested as an effective intervention^[49]^ to manage and cope with stress.^[37]^

Several limitations were noted in this study. First, fibromyalgia patients may have been misclassified in the LHID population because patients in the case cohort with a clinical diagnosis of fibromyalgia as defined by their physicians did not necessarily meet the classification criteria of the American College of Rheumatology.^[50,51]^ However, the larger proportion of female and middle-aged fibromyalgia patients in this study was consistent with previous reports. Moreover, the current study demonstrated a higher standardized prevalence rate (5.84%) and a greater male-to-female ratio in Taiwanese fibromyalgia patients relative to earlier studies.^[1–3]^ This result was similar to that from reports in the United States (6.4%),^[8]^ Germany (5.4%),^[7]^ and Nantou County, Taiwan (6.7%),^[52]^ which used the modified 2010 classification criteria of the American College of Rheumatology to survey the general population. The new criteria, including the widespread pain index and self-reported specific symptoms, may facilitate epidemiological investigations and should help to identify more male and younger patients.^[53]^ Therefore, although the case cohort with a clinical diagnosis of fibromyalgia did not necessarily meet the classification criteria of the American College of Rheumatology, either the 1990 or 2010 version,^[50,51]^ we think the conclusions from this study can be applied to current medical practice given this limitation.

The burden of obesity^[54]^ and benefits of physical activity for patients with rheumatic diseases^[55]^ are well known. Previous reports indicated that overweight or obese women have an increased risk of fibromyalgia, and this was markedly higher for women who were inactive or engaged in less exercise.^[56,57]^ Physical activity is promoted for fibromyalgia patients for stress management,^[49]^ and resilience treatment is recommended to improve SOC.^[58,59]^ However, we were unable to replicate these results because information about the body mass index and physical activity is not provided in the LHID. In addition, the LHID does not provide individual parameters for specific symptoms. Therefore, we could not correlate the degree of pain or other covariates with the risk of a suicide event. As a related limitation, the ICD-9-CM E-codes 950–959 for a suicide event do not correspond to different methods of suicide or self-injury but instead collectively include suicidal behaviors to completed suicide as well as a suicide attempt or nonsuicidal self-inflicted injury, regardless of whether the event resulted in the death of the patient and also regardless of whether the self-injury was driven by suicide ideation.^[60,61]^ Future studies are expected to differentiate between the risk of suicidal behavior and the risk of nonsuicidal self-injury in fibromyalgia patients.

Although the fibromyalgia patients in this study had a higher prevalence of comorbidities than the reference cohort, this association can be attributed to the healthcare-seeking behavior in patients with fibromyalgia. However, we found the risk of a suicide event persisted in patients with primary fibromyalgia relative to subjects with neither fibromyalgia nor comorbidity after adjusting for age and sex in each 2 × 2 stratification analysis with respect to different comorbidities in both fibromyalgia and nonfibromyalgia cohorts; and the risk in fibromyalgia patients with the concomitant comorbidity was markedly enhanced relative to those with primary fibromyalgia. Though one can argue about other residual confounding factors, these confounders are most likely to exist as nondifferential bias with access to such extensive national insurance data and a large sample size. The contention that fibromyalgia is an independent risk factor for suicidal behavior may be strengthened by the evidence described above and should raise concerns among healthcare providers for patients with concomitant comorbidity.

The strengths of this study warrant mention. First, the study data source, Taiwan's NHIRD, enrolls over 22 million citizens, and employees in a national insurance program. The reliability of diagnoses based on insurance claims is strengthened by the stringent NHI surveillance program, which rigorously monitors and audits insurance reimbursement claims to prevent healthcare fraud. Second, the characteristics of our fibromyalgia patients were consistent with previous reports of female predominance and a majority of middle-aged individuals (35–65 years), that is, in the peri-menopausal period.^[1,3,7,8,50]^ The large sample created adequate study power for subgroup analysis to ascertain the impact of fibromyalgia on the risk of a suicide event. Third, the long patient observation period raised the potential to accurately assess the risk of a suicide event in fibromyalgia patients.

In conclusion, our study confirms that fibromyalgia, a chronic pain disorder, increases the risk of suicidal behavior,^[62]^ whereas the risk in patients with primary fibromyalgia is similar to that in patients with other chronic diseases. Fibromyalgia patients with concomitant comorbidity of heart failure, stroke, anxiety, headache, sleep disorder, or depression^[63]^ are associated with a markedly enhanced risk of a suicide event. Even in the absence of a clinical psychiatric disorder, interventions with psychopharmacologic treatment, cognitive-behavior therapy, and aerobic exercise have shown positive effects on fibromyalgia patients’ psychiatric symptoms.^[28]^ Health promotion policies should go beyond health care and strive to eliminate disease risk, which is the objective that the Ottawa Charter 1986 has adopted for the development of health promotion and the maintenance of life.^[36,64]^ It is, thus, of paramount importance to identify fibromyalgia patients with a high suicide risk and provide them with social support and medical treatment to prevent suicidal behavior.

## Acknowledgment

The authors thank the National Health Research Institute in Taiwan for making insurance claims data available for analysis.

## Supplementary Material

Supplemental Digital Content
