# Supplementary material for: Increased risk of a suicide event in patients with primary fibromyalgia and in fibromyalgia patients with concomitant comorbidities: A nationwide population-based cohort study
Source: Medicine (Baltimore). 2016 Nov 4;95(44):e5187. doi: 10.1097/MD.0000000000005187 (PMC5591103; doi:10.1097/MD.0000000000005187)
Supplement: Supplemental Digital Content [file medi-95-e5187-s001.doc]

Supplementary Table 1. Comparison of Suicide Risk between Genders in the Overall Sample Population (n = 856,151) and in Fibromyalgia Cohorts (n = 95,150).

|  | Overall | | Fibromyalgia | |
| --- | --- | --- | --- | --- |
| Variables | Crude HR  (95% CI) | Adjusted HR^1^  (95% CI) | Crude HR  (95% CI) | Adjusted HR^2^  (95% CI) |
| Sex |  |  |  |  |
| Women | 1.10(0.95-1.27) | 1.00(0.86-1.16) | 1.21(0.97-1.50) | 0.96(0.79-1.17) |
| Men | 1.00 | 1.00 | 1.00 | 1.00 |

Adjusted HR^1^: multiple analysis including fibromyalgia, age, occupation, income, comorbidities, and NSAID use.

Adjusted HR^2^: multiple analysis including age, occupation, income, comorbidities, and NSAID use.

| **Supplementary Table 2.** Incidence and Adjusted Hazard Ratio of Suicide in Fibromyalgia Cohort Relative to the Non-Matched Reference Cohort by Stratification with Different Comorbidities | | | | | | | | |
| --- | --- | --- | --- | --- | --- | --- | --- | --- |
|  | Fibromyalgia | | | | | | Compared with reference cohort | |
|  | Yes | | | No | | |
| Variable | Events | PY | Rate# | Events | PY | Rate# | Crude HR  (95% CI) | aHR†  (95% CI) |
| Diabetes |  |  |  |  |  |  |  |  |
| No | 304 | 742,235 | 4.10 | 374 | 1,480,752 | 2.53 | 1.63 (1.40–1.89)*** | 1.40 (1.19–1.64)*** |
| Yes | 43 | 91,263 | 4.71 | 50 | 129,215 | 3.87 | 1.22 (0.81–1.83) | 1.15 (0.76–1.76) |
| Hypertension |  |  |  |  |  |  |  |  |
| No | 242 | 621,194 | 3.90 | 300 | 1,302,455 | 2.30 | 1.69 (1.43–2.01)*** | 1.49 (1.24–1.79)*** |
| Yes | 105 | 212,303 | 4.95 | 124 | 307,512 | 4.03 | 1.23 (0.95–1.60) | 1.09 (0.84–1.43) |
| Hyperlipidemia |  |  |  |  |  |  |  |  |
| No | 287 | 684,041 | 4.20 | 370 | 1,428,307 | 2.59 | 1.62 (1.39–1.89)*** | 1.41 (1.20–1.66)*** |
| Yes | 60 | 149,457 | 4.01 | 54 | 181,659 | 2.97 | 1.35 (0.94–1.96) | 1.14 (0.78–1.66) |
| Heart failure |  |  |  |  |  |  |  |  |
| No | 329 | 814,661 | 4.04 | 403 | 1,584,209 | 2.54 | 1.59 (1.38–1.84)*** | 1.41 (1.21–1.64)*** |
| Yes | 18 | 18,837 | 9.56 | 21 | 25,757 | 8.15 | 1.19 (0.63–2.24) | 0.99 (0.52–1.89) |
| Stroke |  |  |  |  |  |  |  |  |
| No | 288 | 753,614 | 3.82 | 368 | 1,512,008 | 2.43 | 1.57 (1.35–1.84)*** | 1.42 (1.21–1.67)*** |
| Yes | 59 | 79,883 | 7.39 | 56 | 97,959 | 5.72 | 1.31 (0.91–1.89) | 1.15 (0.79–1.68) |
| Depression |  |  |  |  |  |  |  |  |
| No | 281 | 799,670 | 3.51 | 374 | 1,577,599 | 2.37 | 1.48 (1.27–1.73)*** | 1.37 (1.16–1.61)*** |
| Yes | 66 | 33,827 | 19.5 | 50 | 32,367 | 15.5 | 1.29 (0.89–1.86) | 1.25 (0.85–1.83) |
| Anxiety |  |  |  |  |  |  |  |  |
| No | 271 | 754,733 | 3.59 | 369 | 1,541,835 | 2.39 | 1.50 (1.28–1.76)*** | 1.43 (1.21–1.68)*** |
| Yes | 76 | 78,764 | 9.65 | 55 | 68,131 | 8.07 | 1.22 (0.86–1.72) | 1.06 (0.74–1.51) |
| IBS |  |  |  |  |  |  |  |  |
| No | 301 | 763,456 | 3.94 | 388 | 1,535,712 | 2.53 | 1.56 (1.35–1.82)*** | 1.39 (1.18–1.63)*** |
| Yes | 46 | 70,041 | 6.57 | 36 | 74,254 | 4.85 | 1.36 (0.88–2.10) | 1.24 (0.79–1.95) |
| Headache |  |  |  |  |  |  |  |  |
| No | 294 | 791,611 | 3.71 | 403 | 1,580,853 | 2.55 | 1.46 (1.26–1.70)*** | 1.35 (1.16–1.59)*** |
| Yes | 53 | 41,886 | 12.65 | 21 | 29,113 | 7.21 | 1.80 (1.09–2.99)* | 1.62 (0.97–2.72) |
| Sleep disorder |  |  |  |  |  |  |  |  |
| No | 202 | 678,255 | 2.98 | 330 | 1,469,884 | 2.25 | 1.33 (1.12–1.58)** | 1.36 (1.13–1.63)*** |
| Yes | 145 | 155,242 | 9.34 | 94 | 140,083 | 6.71 | 1.40 (1.08–1.82)** | 1.34 (1.03–1.75)* |
| Live cirrhosis |  |  |  |  |  |  |  |  |
| No | 258 | 661,659 | 3.90 | 349 | 1,411,163 | 2.47 | 1.58 (1.34–1.86)*** | 1.40 (1.19–1.66)*** |
| Yes | 89 | 171,838 | 5.18 | 75 | 198,803 | 3.77 | 1.38 (1.02–1.88)* | 1.25 (0.91–1.71) |

PY = person-year, HR = hazard ratio, aHR = adjusted hazard ratio, 95% CI = 95% confidence interval, IBS = irritable bowel syndrome

#Rate, incidence rate (per 10,000 PY).

†The aHR consisted of a multivariate analysis including age, sex, occupation, monthly income, comorbidities, and NSAID use.

‡Anxiety also includes trauma- and stressor-related disorders.

**P* < .05, ***P* < .01, ****P* < .001.

| Supplementary Table 3. The Interaction Relationship between Fibromyalgia and the Respective Comorbidity on the Risk of Suicide Event. | | | | | | |
| --- | --- | --- | --- | --- | --- | --- |
| Variable | | N | Event | Crude HR  (95% CI) | Adjusted HR  (95% CI) | *P^#^* |
| Fibromyalgia | Diabetes |  |  |  |  | .2024 |
| No | No | 173289 | 374 | 1.00 | 1.00 |  |
| No | Yes | 17010 | 50 | 1.52(1.13-2.05)** | 1.51(1.11-2.04)** |  |
| Yes | No | 84233 | 304 | 1.63(1.40-1.89)*** | 1.63(1.40-1.89)*** |  |
| Yes | Yes | 10917 | 43 | 1.86(1.36-2.56)*** | 1.85(1.34-2.55)*** |  |
|  |  |  |  |  |  |  |
| Fibromyalgia | Hypertension |  |  |  |  | .0444 |
| No | No | 150860 | 300 | 1.00 | 1.00 |  |
| No | Yes | 39439 | 124 | 1.74(1.41-2.15)*** | 1.93(1.53-2.44)*** |  |
| Yes | No | 69975 | 242 | 1.69(1.43-2.01)*** | 1.69(1.42-2.00)*** |  |
| Yes | Yes | 25175 | 105 | 2.15(1.72-2.68)*** | 2.37(1.86-3.02)*** |  |
|  |  |  |  |  |  |  |
| Fibromyalgia | Hyperlipidemia |  |  |  |  | .3880 |
| No | No | 168038 | 370 | 1.00 | 1.00 |  |
| No | Yes | 22261 | 54 | 1.14(0.86-1.52) | 1.11(0.83-1.48) |  |
| Yes | No | 77862 | 287 | 1.62(1.39-1.89)*** | 1.62(1.39-1.90)*** |  |
| Yes | Yes | 17288 | 60 | 1.55(1.18-2.04)** | 1.51(1.14-1.99)** |  |
|  |  |  |  |  |  |  |
| Fibromyalgia | IBS |  |  |  |  | .5643 |
| No | No | 180844 | 388 | 1.00 | 1.00 |  |
| No | Yes | 9455 | 36 | 1.90(1.35-2.68)*** | 1.88(1.34-2.65)*** |  |
| Yes | No | 86751 | 301 | 1.56(1.35-1.82)*** | 1.56(1.35-1.82)*** |  |
| Yes | Yes | 8399 | 46 | 2.59(1.91-3.51)*** | 2.57(1.89-3.49)*** |  |
|  |  |  |  |  |  |  |
| Fibromyalgia | Live cirrhosis |  |  |  |  |  |
| No | No | 165786 | 349 | 1.00 | 1.00 | .4498 |
| No | Yes | 24513 | 75 | 1.52(1.19-1.95)*** | 1.52(1.18-1.96)** |  |
| Yes | No | 75301 | 258 | 1.58(1.35-1.86)*** | 1.58(1.34-1.85)** |  |
| Yes | Yes | 19849 | 89 | 2.10(1.66-2.65)*** | 2.10(1.66-2.66)*** |  |
|  |  |  |  |  |  |  |
| Fibromyalgia | Heart failure |  |  |  |  | .3717 |
| No | No | 186127 | 403 | 1.00 | 1.00 |  |
| No | Yes | 4172 | 21 | 3.15(2.03-4.88)*** | 3.17(2.03-4.98)*** |  |
| Yes | No | 92575 | 329 | 1.59(1.38-1.84)*** | 1.59(1.38-1.84)*** |  |
| Yes | Yes | 2575 | 18 | 3.72(2.32-5.96)*** | 3.76(2.32-6.09)*** |  |
|  |  |  |  |  |  |  |
| Fibromyalgia | Stroke |  |  |  |  | .3364 |
| No | No | 176498 | 368 | 1.00 | 1.00 |  |
| No | Yes | 13801 | 56 | 2.33(1.76-3.08)*** | 2.49(1.85-3.34)*** |  |
| Yes | No | 85454 | 288 | 1.57(1.35-1.84)*** | 1.57(1.35-1.84)*** |  |
| Yes | Yes | 9696 | 59 | 3.03(2.30-3.99)*** | 3.22(2.42-4.29)*** |  |
|  |  |  |  |  |  |  |
| Fibromyalgia | Depression |  |  |  |  | .4386 |
| No | No | 186026 | 374 | 1.00 | 1.00 |  |
| No | Yes | 4273 | 50 | 6.46(4.80-8.67)*** | 6.49(4.81-8.74)*** |  |
| Yes | No | 91084 | 281 | 1.49(1.27-1.73)*** | 1.49(1.27-1.73)*** |  |
| Yes | Yes | 4066 | 66 | 8.20(6.31-10.7)*** | 8.23(6.32-10.8)*** |  |
|  |  |  |  |  |  |  |
| Fibromyalgia | Anxiety |  |  |  |  | .2424 |
| No | No | 181568 | 369 | 1.00 | 1.00 |  |
| No | Yes | 8731 | 55 | 3.34(2.52-4.44)*** | 3.37(2.53-4.49)*** |  |
| Yes | No | 85753 | 271 | 1.50(1.29-1.76)*** | 1.50(1.29-1.76)*** |  |
| Yes | Yes | 9397 | 76 | 4.02(3.14-5.14)*** | 4.04(3.15-5.18)*** |  |
|  |  |  |  |  |  |  |
| Fibromyalgia | Headache |  |  |  |  | .4710 |
| No | No | 186648 | 403 | 1.00 | 1.00 |  |
| No | Yes | 3651 | 21 | 2.81(1.81-4.35)*** | 2.76(1.78-4.29)*** |  |
| Yes | No | 90238 | 294 | 1.46(1.26-1.70)*** | 1.46(1.26-1.70)*** |  |
| Yes | Yes | 4912 | 53 | 4.95(3.72-6.60)*** | 4.90(3.67-6.53)*** |  |
|  |  |  |  |  |  |  |
| Fibromyalgia | Sleep disorder |  |  |  |  | .8006 |
| No | No | 172222 | 330 | 1.00 | 1.00 |  |
| No | Yes | 18077 | 94 | 2.96(2.36-3.73)*** | 3.08(2.43-3.89)*** |  |
| Yes | No | 76355 | 202 | 1.33(1.12-1.58)*** | 1.33(1.12-1.58)** |  |
| Yes | Yes | 18795 | 145 | 4.14(3.41-5.03)*** | 4.26(3.49-5.19)*** |  |
| Model adjusted for age and sex; ^#^*P* for interaction; **P*< .05, ***P*< .01, ****P*< .001  HR, hazard ratio; IBS: irritable bowel syndrome; Anxiety also includes trauma- and stressor-related disorders. | | | | | | |
